# Assessment of Malnutrition in Community-dwelling Elderly People: Cooperation among General Practitioners and Public Health

**Published:** 2018-05

**Authors:** Maura FERRARI BRAVO, Fabrizio GALLO, Concetta MARCHELLO, Roberta BOICELLI, Silvia LUPI, Massimiliano ATZEI, Francesco BRUNETTI, Romina CASARETTO, Federico DAPELO, Daniela GEREVINI, Eliana LEONARDELLI, Anna MANNO, Elisabetta PERI, Paola SOAVE, Angelo TRAVERSARO, Antonio ZAMPOGNA, Roberto ZUNINO, Armando STEFANATI, Giovanni GABUTTI

**Affiliations:** 1. Struttura Complessa Igiene e Sanità Pubblica, ASL4 Chiavarese-Regione Liguria, Corso Dante 163, 16043, Chiavari, Italy; 2. Struttura Semplice Dipartimentale Dietetica e Nutrizione Clinica, ASL4 Chiavarese-Regione Liguria, via Terzi 43/A, 16039, Sestri Levante, Italy; 3. Dipartimento di Scienze Mediche, Sezione di Medicina di Sanità Pubblica, Università degli Studi di Ferrara, via Fossato di Mortara 64B, 44121Ferrara, Italy; 4. The Study Group of Società Italiana di Medicina Generale (SIMG), Sezione Tigullio, Italy

**Keywords:** Malnutrition, Free-living elderly people, MNA, Dietetic counseling

## Abstract

**Background:**

Malnutrition, a very common condition in the elderly, is known to increase their vulnerability to adverse health events. This study aimed to estimate the prevalence of malnutrition in the over 75-yr-old community-dwelling population in the “Chiavarese” Local Health Unit district (North West Italy).

**Methods:**

The short version of the Mini Nutritional Assessment (MNA-SF) was used by General Practitioners (GP) as a screening tool to investigate the nutritional status of elderly people (1039 subjects). The study was conducted in 2012–2013 in the Local Health Unit of Chiavari (Liguria Region), Italy. The malnutrition was subsequently confirmed by means of biochemical parameters. Subjects at risk of malnutrition or malnourished (n=22) received personalized dietary counseling by the GP.

**Results:**

The MNA-SF recognized 21% of the elderly people being at risk of malnutrition and biochemical tests confirmed a malnutrition prevalence of 3.5%. The dietary counseling improved the MNA-SF score and biochemical parameters, but the difference was statistically significant only for the MNA-SF score (*P*=0.00613).

**Conclusion:**

Malnutrition can be evaluated with a simple tool such as the MNA-SF, recognized at the earliest stage and successfully treated by the GP.

## Introduction

Malnutrition represents “a state of deficient energy or protein intake or absorption, characterized by weight loss and changes in body composition” (1). It is a very common condition in the elderly population, causing severe adverse health events. Poor nutritional status often presents as unintentional weight loss. When older persons lose weight, they experience a doubling in their risk of death, even if they are overweight (2, 3). The risk increases particularly in people suffering from diseases due to obesity, such as diabetes mellitus. Malnutrition also increases the chance of hip fracture or being institutionalized (4) and represents a significant predictor of frailty syndrome in older persons (5).

Physiological, social and economic issues, often referred as the “nine d’s”, namely poor dentition, dysgeusia, dysphagia, diarrhea, depression, disease, dementia, dysfunction, and drugs, are the main causes (6). The so-called protein-energy malnutrition (PEM) is triggered and, in older people, this has serious consequences including poorer quality of life and greater risk of falls (7,8), an increase in morbidity, mortality and complications of clinical interventions, prolonged length of stay in hospital, admission to higher level care and higher risk of institutionalization with a rise in healthcare costs (9–11). Even though PEM is a common problem, it has not been adequately investigated.

The prevalence of PEM in the elderly varies considerably, depending on the population studied (12), the study setting and the means used to quantify the grade of severity. The settings with the highest levels of malnutrition are nursing homes (up to 85%) and hospitals (up to 62%) (13,14). The risk of malnutrition, instead, affects about half the elderly people receiving home care (15), gradually decreasing until it almost disappears in healthy community-living older adults with increasing degrees of independence (16). Surveys on non-institutionalized elderly subjects have produced highly variable results, depending not only on the criteria used to define malnutrition but also on the geographical areas investigated, although prevalence rates reached significant values up to 30% (7, 17, 18). The studies carried out in Italy have estimated a prevalence ranging from 8% to 12% in community-dwelling persons (19, 20), but significantly higher rates in nursing homes (21, 22).

Management of the severe stage of PEM becomes difficult (23) and early screening in order to verify the nutritional status of the elderly and early preventive intervention in the subjects recognized as being deficient or at risk are therefore crucial tools to ensure good aging. Nutritional status can be investigated using clinical methods, biochemical parameters, anthropometric measurements or multidimensional evaluations; however, none of these alone possesses the ideally necessary requirements (24). Among multidimensional methods, due to its high sensitivity, specificity and reliability, the Mini Nutritional Assessment (MNA) are considered a good and sound method for the evaluation of the elderly population and the detection of subjects with normal nutritional status, and those at risk of malnutrition or malnourished (25). The shorter form (MNA-SF) provides results that correlate well with those of the full version (26), so it can be successfully used for screening, with the advantage of being simpler and faster. The determination of some biochemical parameters (albumin, prealbumin and C-reactive protein) in patients with low scores subsequently makes it possible to confirm the status of malnutrition and to assess the degree of seriousness (27). The plasma concentration of prealbumin allows evaluation of acute PEM, while albumin is an indicator of long-term protein modifications, being significantly reduced only after extended periods of malnutrition. Since both albumin and prealbumin levels are influenced by the inflammatory state, they should be evaluated in relation to the concentration of C-reactive protein (CRP) (28).

The aim of the study was to estimate the prevalence of malnutrition in the population over 75 yr old living at home and resident in the “Chiavarese” Local Health Unit district (North West Italy). The subjects recognized as malnourished or in a condition of risk-received counseling, consisting in a personalized diet recommended by their General Practitioner (GP). The attempt to improve the management of patients with inadequate diet was based on “patient empowerment” in order to change their lifestyle.

## Materials and Methods

### Participants

The study was carried out between Nov 2012 and Dec 2013. The participants were recruited by the general practitioners (GP) of the “Chiavarese” Local Health Unit district (Liguria Region, Italy) who agreed to take part in the study. Individuals aged 75 yr and older, not institutionalized, that spontaneously attended outpatient clinics for therapeutic management or contacted according to the proactive medicine approach were enrolled.

They were informed about the aim of the study and gave written informed consent.

Exclusion criteria were: the absence of the above-mentioned requirements, not receiving the information on the study, the presence of a neo-plastic disease diagnosed less than one year before and/or with clinical signs of activity, severe psychiatric symptoms or which in any case did not allow them to provide informed consent, already activated artificial nutrition (parenteral, enteral), chronic renal failure (creatinine > 3 mg/dl), life expectancy shorter than 6 months.

Since there are no absolute widely accepted criteria for frailty determination, we focused on socio-demographic attributes and components concerning physical and social domains. Accordingly, individuals presenting at least one of these conditions were considered frail: having more than two chronic diseases or a single complicated chronic disease, living alone or in a condition of social disadvantage, having the prescription charge exemption for low income.

### Nutritional assessment

Nutritional status was investigated using the Mini Nutritional Assessment Screening Form (MNA-SF), provided with the software Millewin/MilleGPG used by the GPs. The MNA-SF is the short version of MNA, a tool for nutritional screening specifically designed for elderly people. It consists of six questions that consider recent weight and appetite loss, mobility, acute disease or psychological stress and body mass index (BMI). Each question is scored from zero to two or three, for a maximum score of 14. A score of 12 and above indicates satisfactory nutritional status; a score ranging from 8 to 11 suggests a risk of malnutrition and a score of 7 and below shows a malnutrition status (26). In subjects with a score up to 11, plasma levels of prealbumin, albumin and CRP were assessed. Albumin and prealbumin are commonly used indicators for identification and classification of malnutrition, while CRP makes it possible to control possible confounding effect of inflammatory status. We measured prealbumin and albumin with a nephelometric assay (BNII, Laser Nephelometry, Dade Behring). CRP was evaluated by turbidimetric method (Modular PP, Roche). All the procedures were in accordance with the Health Quality Service Standards. The degree of malnutrition in patients with MNA scores under 12 was classified according to the algorithm shown in Table 1.

**Table 1:** Algorithm for malnutrition assessment

***Albumin***	***Prealbumin***		
	≥17 mg/dl	10–17 mg/dl	<10 mg/dl
≥3000 mg/dl	Possible recent recovery of good nutritional status	Recent mild PEM	Recent severe PEM
<3000 mg/dl	PEM in recent improvement	Chronic mild PEM	Chronic severe PEM

Developed and adapted from (29)

### Nutritional counseling

Subjects with an MNA score ≥ 12, not presenting malnutrition, received information on how to maintain an adequate nutritional status and suggestions for the continuation and improvement of a suitable lifestyle. Tailored dietary counseling correlating the degree of malnutrition and the most appropriate dietary indications according to the characteristics of the subject (gender, weight, chewing ability, independence, purchasing skills) was recommended by the GP to the patients with an MNA score under 12 and PEM confirmed by biochemical parameters. In the case of impaired chewing ability, a soft diet was suggested. The selection of patients needing dietary advice was made by the GPs with the collaboration of the Nutrition Service of the Local Health Unit that prepared the diets. Two months after the implementation of the suggested dietary regime, a further assessment of nutritional status with the MNA-SF and an additional evaluation of plasma biochemical parameters were performed.

### Ethical aspects

The study received the approval of the Local Health Unit Ethics Committee according to current legislation. The collection of data and biological samples was conducted in compliance with the protection of personal data.

### Statistical analysis

MNA-SF score, albumin and prealbumin concentrations expressed as mean ± standard deviation were compared between genders with the Student *t*-test. The multivariate regression analysis (adjusted for gender and age as confounding factors) was applied to evaluate, before and after treatment, the variations in biochemical parameters and MNA-SF score corrected for the confounding effect due to CRP. Statistical analysis was performed with R (R free software, ver. 3.0.3) and the significance set at 0.05.

## Results

Eleven GPs participated in the study, involving 1039 over 75-yr-old community-dwelling persons. Complete data are available for 821 of them, representing a participation rate of 79%. The main demographic characteristics are reported in Table 2. The average age of enrolled patients was 82 yr; 65% were women.

**Table 2:** Demographic characteristics of the enrolled elderly people

***Variable***	***Females n (%)***	***Males n (%)***	***Total n (%)***
	537 (65.4)	284 (34.6)	821 (100)
Age. (mean ±s.d, yr)	82±6.1	82±4.9	82±5.7
Frailty	520 (96.8)	266 (93.7)	786 (95.7)
Due to disease	446 (83.1)	230 (80.1)	676 (82.3)
Living alone	234 (43.6)	47 (16.6)	281 (34.2)
Social disadvantage	28 (5.2)	6 (2.1)	34 (4.1)
Prescription charge exemption	431 (80.3)	197 (69.4)	628 (76.5)
MNA-SF score (mean ±s.d.)	12.1±2.1	12.7±1.7	12.3±2.0
0–7	24 (4.5)	4 (1.4)	28 (3.4)
8–11	124 (23.1)	48 (16.9)	172 (21.0)
12–14	389 (72.4)	232 (81.7)	621 (75.6)

Almost all the participants (95.7%) could be considered frail individuals, mainly due to the presence of illness conditions and the prescription charge exemption for low income. The nutritional evaluation by means of the MNA-SF showed that 21% of elderly people living in the community were in a situation of risk of malnutrition and the prevalence of malnutrition was 3.4%. Both conditions were more prevalent in the females (respectively 23.1% and 4.5% versus 16.9% and 1.4% in males).The determination of biochemical parameters for confirmation of the malnutrition status made it possible to estimate a prevalence of 3.5% (Table 3). Malnutrition was again found to be more common in females, although no statistically significant difference was observed between the two genders. The majority of malnourished subjects (69%) achieved an MNA-SF score between 8 and 11, only indicative of a risk of malnutrition. The average levels of the biochemical parameters were suggestive of PEM in recovery for men and recent mild PEM for women.

**Table 3:** Levels of albumin and prealbumin in patients with MNA-SF <12 and malnutrition confirmed by biochemical parameters (n = 29) according to gender and MNA-SF score (mean±s.d.)

***n, %***	***MNA-SF score***	***Prealbumin (mg/dl)***	***Albumin (mg/dl)***
Females (17, 58.6%)	7.8±2.9	14.8±3.2	3421.5±585.9
Males (12, 41.4%)	9.0±1.7	17.8±5.6	3189.9±608.4
*P*	0.2142	0.0788	0.3113
MNA-SF score 0–7 (9, 31.0%)		13.2±4.0	3061.1±718.9
MNA-SF score 8–11 (20, 69.0%)		17.8±5.6	3444.7±507.5
Total		16.0±4.5	3325.7±595.9

The differences between genders were assessed with Student t-test

**Fig. 1:**
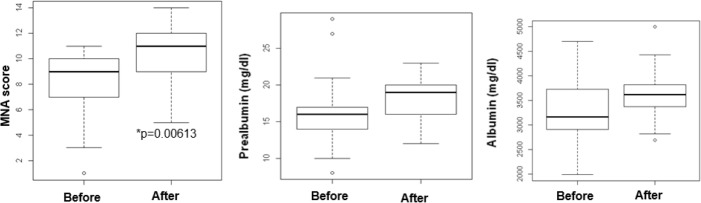
Characteristics of patients with MNA-SF <12 and malnutrition confirmed by biochemical parameters before and after counseling (n = 22) The statistically significant difference of MNA score, before and after counseling, was determined by regression analysis corrected for the CRP

A significant proportion (75%) of patients with malnutrition confirmed by blood parameters received tailored dietary counseling. All measured parameters had improved, as showed by median, first, and third quartiles reported in Fig. 1.

The regression analysis corrected for the CRP showed that dietary intervention improved all considered parameters but the difference was statistically significant only for the MNA-SF score (*P*=0.00613).

The mean MNA-SF reached an average score of 11. Only two subjects remained in the range of malnutrition, despite improving their score, while 8 individuals scored over 12, showing recovery of a normal nutritional status (data not shown). The levels of albumin and prealbumin increased and the latter, in particular, exceeded the threshold of 17 mg/dl, indicating a recent recovery of a good nutritional status.

## Discussion

The elderly population is particularly vulnerable to malnutrition. Although this condition is more frequent among institutionalized persons, prevalence rates not entirely insignificant and varying according to the degree of independence have also been reported among older people living in the community. Western countries are characterized by a progressive increase in average life expectancy but this implies a higher prevalence of adverse health events. These are strongly associated with malnutrition and include reduced quality of life, increased complications of diseases, higher risk of institutionalization, greater frailty and increased mortality. More attention should be directed at malnutrition, a condition often unrecognized and underestimated.

Compared to the Italian population in general, the inhabitants of the “Chiavarese” Local Health Unit district are characterized by a lower number of young people and of the so-called active population, while the elderly component is predominant. With respect to the Italian population in general, there are more than one and a half times more 64-yr-old subjects in this area and more than double over 84-yr-old (4% vs. 2%). The Ligurian population is, in fact, the oldest of Italy and the residents of the “Chiavarese” Local Health Unit district are the eldest of Liguria. Since the demographic transition that already occurred in this area prefigures the transition that the Italian population and those of all economically advanced countries will undergo in the coming decades, this district and its population can represent a test case for assessing and proposing solutions to the health and social problems of an increasingly elderly community.

This study made it possible to estimate the prevalence of malnutrition in the over-75-yr-old population cared for by GPs. 3.5% of the enrolled elderly showed a malnutrition status confirmed by biochemical parameters. Due to the variations depending on the method adopted, it is very difficult to compare this finding with those reported by other studies. However, the acquisition of updated epidemiological data on the malnutrition status of the elderly population is a basic requirement for the definition of programs aiming to control and prevent malnutrition. The MNA-SF proved to be a reliable, simple and easy tool to detect the risk of malnutrition in elderly people living in the community. The adoption of a standardized methodology, available to GPs, can contribute to the timely monitoring of the nutritional status of older patients, and the early identification of deficiency situations may allow effective recovery actions to be implemented.

Previously, the Nutrition Service of this district had already carried out nutritional surveillance in hospitalized patients both in the hospital setting (29) and in protected structures (21). This study also represents the first experience of personalized nutritional counseling, led by GPs in agreement with the Nutrition Service, aimed at improving the nutritional status of their patients. In addition to the evaluation of malnutrition, the surveillance provided information for the preservation of nutritional status for elderly with an appropriate MNA score and provided the dietary indications for people with different levels of malnutrition according to body weight and chewing ability. The interplay and coordination between GPs, Nutrition Service and Hygiene and Public Health guaranteed a favorable multidisciplinary approach that made it possible to construct a healthcare pathway consisting of tailored dietary counseling that was able, in two months, to improve the nutritional status and the related biochemical parameters in the elderly living in the community. Previous experiences of nutritional intervention in the elderly population in Europe have produced conflicting results. A study in the Netherlands (30) showed no result in terms of improvement in body weight, physical performance and handgrip strength at 6 months after nutritional counseling by dieticians. After one year, an increase of 2.5 points was found in the MNA score in a group of elderly individuals given tailored nutritional counseling by a dietician and counseling for physical activity by a physiotherapist (31). Older people who received the counseling also improved their frailty status and obtained a higher Mini-Mental State Examination (MMSE) score, indicative of better cognitive function compared to the control group in which the MNA score worsened.

Although the study involved a limited number of GPs and over 75-yr-old subjects, it made it possible to explore the problem of malnutrition in the elderly living in the community, based on a methodology previously established through two observational studies conducted in hospitals and in protected structures (29, 21). In addition, the investigated population reflects demographic features that will characterize all Western societies at the end of the process of demographic transition. It can be taken therefore, as a paradigm for the testing of actions aimed at promoting healthy aging.

## Conclusion

The GP, a valuable reference point for elderly patients, can monitor and recognize the malnutrition status or related risk early, thus successfully implementing personalized intervention, in order to achieve regression of the PEM, as shown by the improvement of the MNA-SF score and biochemical parameters. This finding may have been influenced by the fact that the patients received customized dietary indications and were motivated to change their lifestyle by a trusted person like the GP; in addition, the participants in our study presented only a mild PEM status. Given the positive results obtained with a follow up of only two months, we can hypothesize that the extension of the “empowerment” intervention could encourage the patient to continue to follow the dietary recommendations over time, consolidating the result and ensuring a better quality of life.

Malnutrition can be assessed with a simple tool such as the MNA-SF, recognized at the earliest stage and successfully treated by GPs, as shown by the increase by two points of the score recorded in patients receiving a personalized diet. This result has important and promising implications in the management of the older population, as most of them live in the community and could benefit considerably from nutritional counseling. The improvement of the nutritional status of community-dwelling people can, in fact, constitute an effective method of prevention of adverse health events such as hospitalizations, complications, readmissions, institutionalization and mortality.

## Ethical considerations

Ethical issues (Including plagiarism, informed consent, misconduct, data fabrication and/or falsification, double publication and/or submission, redundancy, etc.) have been completely observed by the authors.
